# Association between Access to Electronic Devices in the Home Environment and Cardiorespiratory Fitness in Children

**DOI:** 10.3390/children6010008

**Published:** 2019-01-09

**Authors:** Christopher D. Pfledderer, Ryan D. Burns, Timothy A. Brusseau

**Affiliations:** Department of Health, Kinesiology and Recreation, University of Utah, Salt Lake City, UT 84112, USA; ryan.d.burns@utah.edu (R.D.B.); tim.brusseau@utah.edu (T.A.B.)

**Keywords:** physical fitness, sedentary behavior, screen-time, children

## Abstract

This study examined the association between access to electronic devices in the home and cardiorespiratory fitness in children. Participants were children aged 8–12 years from a local elementary school (*n* = 106, mean age = 9.7 + 1.1 years, male = 50). Child access to electronic devices was measured with a 37-item parent-reported questionnaire. Estimated maximal aerobic capacity (VO_2 Peak_) was calculated from The Progressive Aerobic Cardiovascular Endurance Run (PACER) using a validated algorithm. The association between access to electronic devices in the home and cardiorespiratory fitness was explored by employing hierarchical ridge regression, using the Ordinary Least Squares (OLS) model, controlling for the covariates of sex, age, and Body Mass Index (BMI). Controlling for sex, age, and BMI, the number of electronic items in a child’s bedroom was significantly inversely related to the estimated VO_2 Peak_ (b = −1.30 mL/kg/min, 95% C.I.: −2.46 mL/kg/min, −0.15 mL/kg/min, *p* = 0.028) and PACER laps (b = −3.70 laps, 95% C.I.: −6.97 laps, −0.41 laps, *p* = 0.028) However, the total number of electronic items in the home and total number of electronic items owned did not significantly relate to the estimated VO_2 Peak_ (*p* = 0.847, 0.964) or the number of PACER laps (*p* = 0.847, 0.964). Child health behavior interventions focused on the home environment should devote specific attention to the bedroom as a primary locus of easily modifiable intervention.

## 1. Introduction

It has been nationally [1] and globally [2] established that children should accumulate at least 60 min of moderate to vigorous physical activity per day and should be exposed to no more than 2 h of screen-time per day [3]. A lack of physical activity has been shown to be positively associated with a wide range of adolescent health issues including cardio-metabolic risk factors [4,5], adiposity [6], depression and sleep problems [7], and obesity [8]. Moreover, habitual inactivity, in the form of screen-time and lack of physical activity, carries over into adulthood [9,10]. Regardless of these recommendations, less than four out of 10 American elementary school-aged children meet both physical activity and sedentary behavior standards concurrently [11], with nearly 47% spending more than two hours per day engaging in sedentary, screen-based activities [12].

While the use of screen-based, electronic media can occur in many locations, the home environment represents a primary location for this use to occur among children. One reason is because children have less independence and fewer means to travel longer distances from the home compared to adolescents (e.g., lack of driver’s license, earlier curfews). Numerous studies have explored the in-home microscale environment and its impact on electronic media use among children. The majority of these home-based studies have found significant, positive associations between both the number of electronic devices and/or the ease of access to electronic devices in the home and screen-time in adolescent populations [13,14]. These findings align with current ecological models of health behavior, which highlight the home environment and the electronic equipment that is present in the home environment as one facet of health behavior intervention [15].

One specific area of the home that represents a particularly important and frequent location of the use of screen-based media among children and adolescent populations is the bedroom. Studies have shown that access to screens and other electronic media, specifically in the bedroom, is associated with a number of detrimental effects, including higher sedentary time [16] and screen-time [17], lower reading time and higher BMI, waist circumference, and body fat [18], less time spent in vigorous physical activity, lower fruit and vegetable intake, and lower grade point average [19]. Another important and frequently studied aspect of in-bedroom screen-time is its effect on the quantity and quality of sleep among children and adolescents. A systematic review of screen-time and sleep among school-aged children found that screen-time was adversely associated with both the quality and quantity of sleep in 90% of the studies reviewed [20]. Other studies have produced similar results, including associations between in-bedroom screen-time and short sleep duration, increased sleep deficiency [21], and daytime sleepiness [22].

However, the majority of studies that focus on children and adolescents’ home environment, which include access to and use of electronic media, are primarily concerned with its effect on general physical activity [23,24,25]. Few studies have explored the relationship between the micro-scale home environment, access to electronic media, and health-related fitness markers among children. Those that have explored this relationship have used obesity or Body Mass Index (BMI) as the primary outcome of interest and have found significant associations [22,26,27].

While body composition measurements are important indicators of overall health, other important markers exist as well, including cardiorespiratory fitness (CRF). A number of studies have shown significant associations between increased CRF in children, typically assessed as maximal aerobic capacity (VO_2 peak_), and a variety of positive attributes, including academic achievement [28], cognitive control [29], language processing [30] superior cortical brain structure and increased cerebral blood flow [31,32], lower risk of high blood pressure [33], and lower risk of death from any cause later in life [34].

To the authors’ knowledge, no study has explored the relationship between access to electronic devices in the home environment and CRF among elementary-aged children. Given the strong influence of the home environment on children’s electronic device use, the associations between increased electronic device use and a variety of detrimental health and performance outcomes, the positive associations between CRF and a number of beneficial outcomes, and the scarcity of research exploring the effect of electronic device prevalence in micro-scale environments such as the home on aerobic fitness, it is important to further explore this interaction. Therefore, the purpose of this study is to determine the associations between in-home and in-bedroom electronic device access and estimated VO_2 Peak_ among elementary-aged children.

## 2. Materials and Methods

### 2.1. Participants and Setting

Participants were 3rd–6th grade 8–12-year-old students (*n* = 106, male = 50) at a K-6 school located in a metropolitan area of the Southwestern United States. A total of 120 students were invited to participate. Consent for participation in this study, which included aerobic fitness testing and height and weight measurements, was obtained from parents via an online questionnaire. Child assent was also obtained via physical signatures. This study was approved by the university’s Institutional Review Board and the district’s research board prior to the start of the study (IRB_00105987).

### 2.2. Instruments/Measures

#### 2.2.1. Screen-time and Access to Screens

Adolescent screen-time and access to screens was measured with a 37-item online questionnaire administered via Google Forms and was completed by at least one parent of the adolescent. The online questionnaire was adapted from a previously validated hard-copy questionnaire used to assess physical activity and sedentary equipment in the home [35]. Questions irrelevant to sedentary behavior/screen-time were omitted. Only questions related to sedentary behavior/screen-time were used in the version administered for this study, which were Section A and Section R in the original survey.

#### 2.2.2. Health Related Fitness Assessment

Cardiorespiratory fitness was assessed aerobically by using the Progressive Aerobic Cardiovascular Endurance Run (PACER) test, a multistage fitness test adapted from the 20-m shuttle run and used as the default aerobic capacity test in the FITNESSGRAM assessment system. Students practiced the PACER test to familiarize themselves with the procedure in the weeks leading up to the actual test to ensure they performed the test properly on their actual test day. The PACER was conducted on a marked gymnasium floor with background music provided by a compact disk. Each student was instructed to run from one floor marker to another floor marker across a 20-m distance within an allotted time frame. The allotted time given to reach the specified distance incrementally shortened as the test progressed. If the student twice failed to reach the other floor marker, the test was terminated. Results of the PACER test were hand-recorded using the FITNESSGRAM PACER Test Individual Score and PACER Group Score Sheet. This process was repeated for 4 weeks for each specific grade level (3rd grade–6th grade), so that data could be collected on participants in each grade level. The final score was recorded in laps but then converted to estimated VO_2 Peak_ using a validated prediction algorithm [36]. The PACER test has been shown to be both a reliable and valid health assessment [37]. Students completed the PACER test indoors during their regularly scheduled Physical Education class.

#### 2.2.3. Body Mass Index

Body Mass Index (BMI) was calculated using standard procedures by taking a student’s weight in kilograms divided by the square or his or her height in meters. Height was measured to the nearest 0.01 m using a portable stadiometer (Seca 213; Hanover, MD, USA), and weight was measured to the nearest 0.1 kg using a portable medical scale (BD-590; Tokyo, Japan). Height and weight were collected in a private room connected to the school’s gymnasium during each student’s physical education class.

### 2.3. Procedures

Data were collected on students in grades 3–6 during a 4-week period in the spring of the 2017–2018 school year. During the week of assessment, adolescents’ screen-time/access to screens, and aerobic fitness were measured using the previously described measurement techniques. The questionnaire quantifying adolescent screen-time and access to screens was sent to the adolescents’ parents via Google Forms at the beginning of the week before their child’s aerobic fitness was measured during Physical Education class. The survey instructed parents to submit the form electronically by Friday of that same week. This was to ensure adequate time for consent to be given. On the following Monday, adolescents for which consent was given and assent was obtained had their height and weight measured and performed the PACER test as previously described. Data from the questionnaire, PACER scores, and height and weight measurements were compiled and used for analysis.

### 2.4. Statistical Analysis

Data were screened for outliers using z-scores (using a ±3.0z cut-point) and checked for Gaussian distributions using k-density plots. No severe outliers were detected and the distribution of the estimated VO_2 Peak_ (the dependent variable) was approximately Gaussian. Differences between the sexes on all observed variables were examined using independent t-tests. To examine the linear relationships between the number of personal electronic items (PE Composite), personal items in the bedroom (IB Composite), and personal items in the home (IH Composite) with estimated VO_2 Peak_, hierarchical ridge regressions were employed using the Ordinary Least Squares (OLS) model. Ridge regressions were employed to account for potential multicollinearity among predictor variables. Ridge regressions were carried out using STATA’s “ridgereg” command with the OLS model option. A hierarchical predictor variable entry method was employed to examine the independent association of electronic items (Block #1) and the statistical control of pertinent covariates consisting of sex, age, and BMI (Block #2). Separate models were fit for estimated VO_2 Peak_ and PACER laps outcomes. The OLS assumption of heteroscedasticity was examined in the final models using residual vs. fitted plots and statistically tested using the Breusch–Pagan test. The alpha level was set at *p* < 0.05, and all analyses were carried out using the STATA v15.0 statistical software package (College Station, TX, USA).

## 3. Results

**Figure 1:**
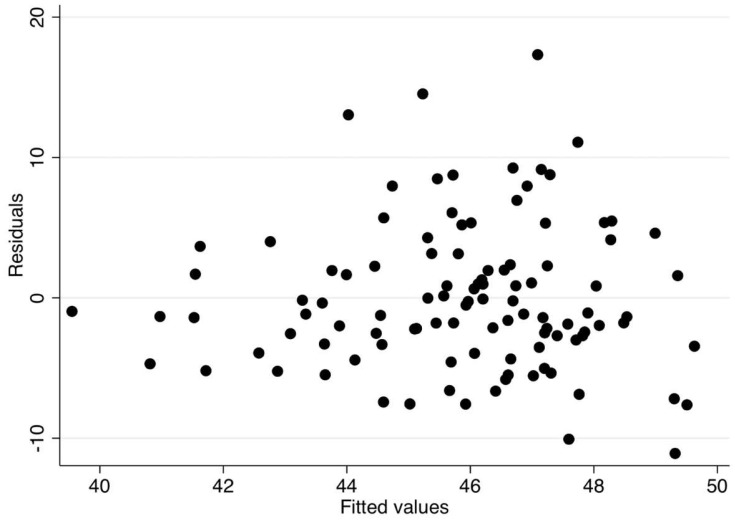
Residual vs. fitted plot showing a heteroscedastic pattern across the range of fitted VO_2 Peak_ scores.

**Figure 2:**
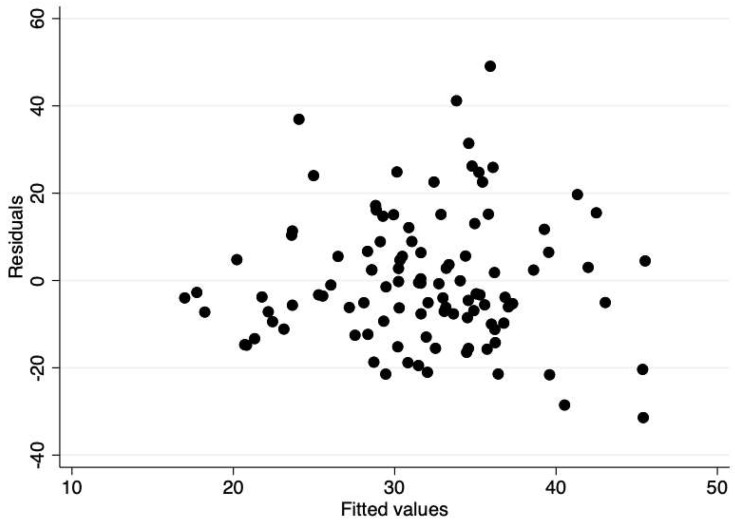
Residual vs. fitted plot showing a heteroscedastic pattern across the range of fitted PACER lap scores.

**Figure 3:**
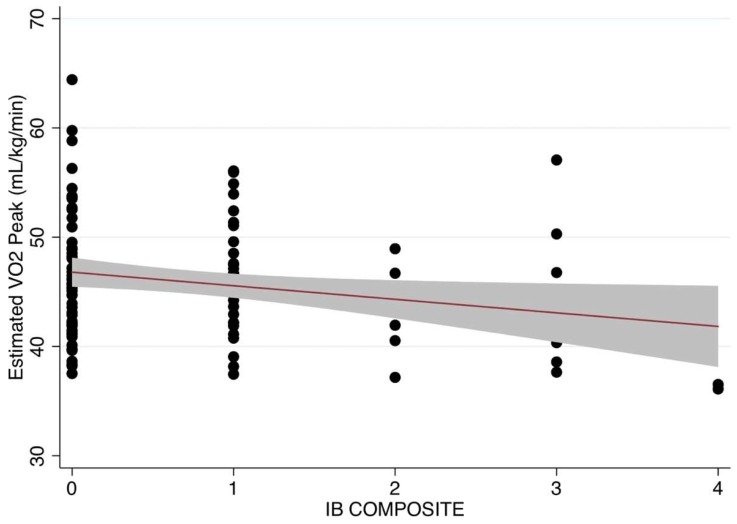
Scatterplot and line of best fit showing the linear relationship between the estimated VO_2 Peak_ and the number of electronics in the bedroom (IB Composite).

**Figure 4:**
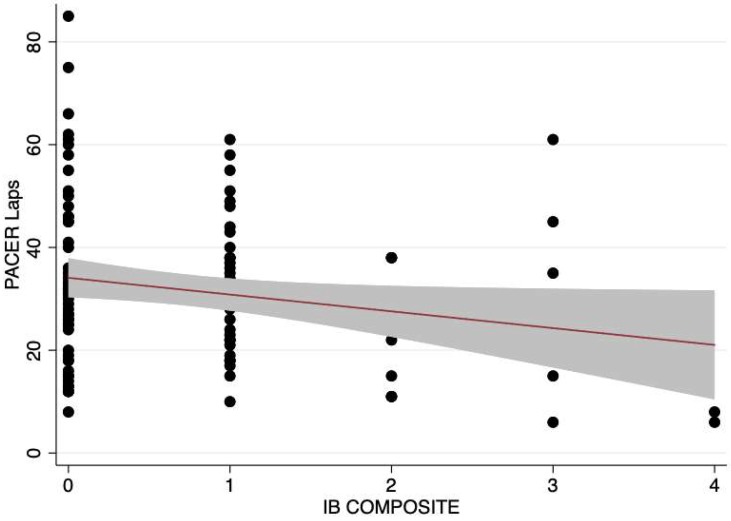
Scatterplot and line of best fit showing the linear relationship between PACER Laps and the number of electronics in the bedroom (IB Composite). Note: PACER stands for the Progressive Aerobic Cardiovascular Endurance Run.

The descriptive statistics for the total sample and within sex groups are communicated in Table 1. There were no statistical differences between sexes on any observed variable (*p* > 0.05). The results from the hierarchical linear ridge regression model are reported in Table 2. The number of electronic items in the bedroom (IB Composite) was significantly related to both the estimated VO_2 Peak_ and PACER laps. This relationship held after controlling for pertinent covariates (i.e., the addition of Block #2). BMI was the only covariate that was significantly related to the estimated VO_2 Peak_ and PACER laps. The residual vs. fitted plots were derived from the final models (Block #1 + Block #2) to inspect heteroscedasticity (Figure 1 and Figure 2). The residual vs. fitted plot showed a heteroscedastic pattern across the range of fitted scores, supporting the assumption for both outcomes. This was statistically supported by a non-significant Chi-square statistic from the Breusch–Pagan tests (estimated VO_2 Peak_: χ^2^(1) = 2.43, *p* = 0.119; PACER laps: χ^2^(1) = 2.94, *p* = 0.081). The predicted residuals also approximated a Gaussian distribution. Figure 3 and Figure 4 visually depict the linear relationship between IB Composite with estimated VO_2 Peak_ and PACER laps, respectively.

## 4. Discussion

The purpose of this study was to determine the associations between in-home and in-bedroom electronic device access and estimated VO_2 Peak_ among elementary-aged children. The current study found a significant negative linear relationship between the number of electronic devices children have in their bedroom, PACER laps, and their estimated VO_2 Peak_ from PACER scores, but not a significant relationship between the number of overall electronic devices found in the home or the overall number of children’s personal electronic devices and their PACER laps or estimated VO_2 Peak_. These findings suggest that the bedroom is an important area of the home in which children should not have a large number of electronic devices, regardless of how many devices exist elsewhere in the home’s micro-scale environment.

Numerous studies have explored the relationship between screen-time and CRF in similarly-aged populations. Since it is presumable that increased access to electronic devices in the bedroom environment leads to an increase in screen-time, this association is an important one to consider. For example, in a study analyzing associations of CRF with physical activity, adiposity, and screen-time, Aires et al. [38] found that screen-time was negatively associated with CRF. It is worth noting that a similar 20-m shuttle run was used to assess CRF in this large sample of children and adolescents. Arango et al. [39] also found significant links between screen-time, CRF, and adiposity in another sample of youths, with the direction of the association being the same as it was in our study. Screen-time, in this case, encompassed not only TV viewing, but also personal computer/video game use. Our study used a similar composite of multiple types of electronic devices. Finally, in a study of 7466 10–16-year-old school children, Sandercock and Ogunleye [40] found that screen-time in both boys and girls was negatively associated with fitness after controlling for physical activity (PA) levels. The authors concluded that their results do indeed support the recommendation to limit screen-time to no more than two hours per day.

Besides CRF, a number of studies have explored the relationship between screen-time and measures of obesity. Laurson et al. [41] found children who met PA and screen-time recommendations were the least likely to be overweight. The authors concluded that children who did not meet these guidelines were three to four times more likely to be overweight when compared to those children who did meet the guidelines. A study exploring the relationship between screen-time and metabolic syndrome in children and adolescents found that weekend screen-time was independently associated with an increased prevalence risk of metabolic syndrome in the sample population [42]. A similar study found an increased risk of insulin resistance among adolescents who did not meet screen-time guidelines during weekdays [43]. Given this evidence and the fact that these results support our results, the exact relationship between CRF, obesity, and electronic device use is important to understand. One explanation is that in-bedroom screen-time displaces PA time in children. In other words, the more time children spend using electronic devices, the more sedentary time they accrue, which results in less time throughout the day to accrue recommended PA levels. This decrease in PA may lead to increased BMI and decreased CRF, which could explain the relationship between in-bedroom electronics and decreased CRF in our study. While these studies are similar to ours, they do not account for where this screen-time is occurring. Similar to our study, Wethington et al. [44] explored screen-time and obesity in relation to the presence of a television in the bedroom. It was found that having a bedroom TV was associated with both increased screen-time and obesity. These results support our study, as it was found that the mere presence of in-bedroom electronics, including TVs, was significantly associated with a measure of CRF. More specifically, an increase in the number of in-bedroom electronics was associated with a decrease in CRF. Considered together, these results could have further implications with regard to sleep, since the bedroom was the primary location of interest.

The majority of studies focusing on electronics in the bedroom explored sleep-related outcome variables. Of studies focused on the relationship between in-bedroom electronic use and sleep, the overwhelming majority found the use of screens before bedtime to be detrimental to both the quantity and quality of sleep in children. The underlying mechanisms of the connection between screen use before bed and sleep in children and adolescents have been well-researched, but not completely realized. Some explanations include the effect of exposure to light on decreased natural melatonin levels [45], which has been shown to be twice as high in children compared to adults [46], the effect of time displacement that the use of electronic media causes, and the effects of psychological arousal that might result from the use of screen-based media [20]. It could be that the mere presence of an electronic device in the bedroom makes children more likely to use it immediately before sleep, resulting in poorer sleep quality and quantity. Another explanation could be that sleep gets interrupted by lights or sounds caused by the electronic device, like receiving a text message or a loud event occurring on a TV show or movie. For example, Adachi-Mejia et al. [47] found that out of 454 adolescents surveyed, 62.9% took their phone to bed, 56.8% kept it turned on while sleeping, 36.7% texted after going to bed, and 7.9% were awakened by a text after falling asleep. Another explanation could be that simply having the device in the bedroom increases arousal before sleep, resulting in a delayed onset of sleep. For example, children who have a cell phone in their bedroom may anticipate a text message, which could result in a delayed onset of sleep as well. Regardless of the mechanism, the body of research on screen-based media use in the bedroom and sleep quality in children almost always concludes that electronic device use should be limited if children are to achieve a healthy amount of sleep. Accruing an appropriate amount of sleep that is of good quality is important for a number of reasons, but for the purposes of this study, sleep may play a mediating role between electronic devices in the bedroom and cardiorespiratory fitness.

The use of screen-based electronics in the bedroom, which has been connected to poor sleep, has also been shown to subsequently lead to a variety of detrimental health-related outcomes. While our study did not measure the actual usage of electronic devices in the bedroom, results still showed a significant relationship between the presence of those devices in adolescents’ bedrooms and their estimated VO_2 Peak_. Lemola and colleagues [48] found that smartphone use in bed before sleep was significantly related to reduced sleep duration and increased sleep difficulty, which mediated the relationship between the use of electronics in the bedroom and depressive symptoms in the adolescent study population. Another study focusing on the longitudinal effects of mobile phone use in the bedroom within an adolescent population found a direct association between increased nightly phone use in the bedroom and externalizing behavior and decreased coping and self-esteem [49]. In another study of adolescent sleep patterns and night-time technology use, Gamble et al. [50] found a dose-response relationship between the use of electronic devices in the bedroom and sleep patterns, which resulted in delayed sleep-wake schedules. The authors concluded that these obstructed patterns could result in a variety of detrimental health and educational outcomes. Finally, a systematic review of the relationship between sleep duration and health outcomes in children found that decreased sleep duration was associated with higher adiposity, lower emotional regulation, and lower quality of well-being [51]. The review also found an association between sleep duration and cardiometabolic markers, although the quality of the majority of these studies was rated low by the authors due to various elements of the study design. Although our study did not assess any sleep-related outcome variables within the population, it is possible that the observed relationship between the larger number of electronic devices in the bedroom and lower estimated VO_2 Peak_ is an indirect result of reduced sleep quality and quantity brought on by increased access to a variety of electronic devices. In other words, sleep may mediate the relationship between the number of electronic devices in a child’s bedroom environment and cardiorespiratory fitness.

The results of our study and the connections between electronic device use, poor sleep outcomes, and decreased CRF are supported by a number of other studies. Although none have used CRF as a primary outcome variable specifically in relation to electronic device access in the bedroom, others have explored the relationships between CRF variables with sleep variables and other health markers. In a cross-sectional study exploring the relationships between sleep quality, cardiorespiratory fitness, and BMI in 1726 adolescent girls, Mota and Vale [8] found that poor sleep quality was significantly associated with lower CRF. Similar to our study, the FITNESSGRAM shuttle run was used to measure cardiorespiratory fitness as an estimated VO_2 Peak_. Chaput et al. [52], using parent-reported data on a large sample of 9–11-year-olds, found that children, regardless of sex, who had 2–3 screens in their bedroom had both a higher body fat percentage and a lower sleep efficiency. However, the authors did not find a significant relationship between the number of screens in the bedroom and the level of moderate-to-vigorous physical activity (MVPA) or sedentary time, which has been linked to measures of estimated VO_2 peak_ in children [53]. Another supporting study found that children who had a television in their bedroom were more likely to have unhealthier diets and higher body fat percentages [54]. Finally, a study of 2334 5th graders found that the odds of obesity doubled in children who had bedroom access to televisions and computers [55]. Similar to the current study, the authors measured access to screens with parent reports.

The relationship between access to electronic devices in the bedroom and children’s estimated VO_2 Peak_ found in our study, in conjunction with the relatively large body of research that has related electronic device use to poor sleep and health outcomes in children, leads to a number of explanations. It could be that access to electronic devices specifically in the bedroom results in an increased use of these devices before bedtime, resulting in poorer sleep quality and/or quantity, which has been shown to result in detrimental health-related outcomes. Because these devices are located in the bedroom, children have easy access to them before and during bedtime and may be more inclined to use them while they are preparing to fall asleep. It could also be that the mere presence of electronic devices in the bedroom leads to poorer sleep, due to interruptions or anticipation, which, in turn, leads to a decrease in health or fitness outcomes in children. Regardless of the underlying mechanisms behind these associations, the results of this study support the idea that it is important to limit children’s access and use of electronic devices in the bedroom, specifically during bedtime.

To the authors’ knowledge, this is the first study to explore the associations between access to electronic devices in the home micro-environment and a measure of CRF in children. This information could help fill gaps in understanding related to the associations of screen-time, sleep quality and quantity, and specific health-related outcomes in children. However, our study was not without limitations. Measurements were taken on a relatively small sample of children and the cross-sectional nature of the study design did not permit us to assume causation between any of the variables. Furthermore, all electronic device access measurements were proxy-reported by parents of the children. Some electronic devices present in children’s bedrooms could have been missed because of this. This study also did not measure actual screen-time or sleep-related variables, which could have provided useful information on the possible connections between electronic device use, sleep, and CRF. Finally, the FITNESSGRAM shuttle run, which was used as an assessment of CRF in our study, is only an approximation of VO_2 Peak_, and is not considered the “gold standard” for VO_2 Peak_ measurements.

## 5. Conclusions

This study found a significant relationship between the number of electronic devices located specifically within children’s bedrooms, PACER laps, and their estimated VO_2 Peak_. However, there were no significant associations between PACER laps, VO_2 Peak_, or the number of electronic devices in any other location of the home. These results suggest that the child’s bedroom is a particularly important location in which to limit the use and access of electronic devices. Interventions aimed at limiting children’s screen-time before bed should put specific emphasis on decreasing the number of electronic devices within the bedroom. It is suggested that future research and interventions pay particular attention to the bedroom as a significant area of the home in which electronic device use should be monitored and subsequently curtailed. Future research should also further explore the relationship between electronic device use/access in the bedroom, sleep metrics, and measures of fitness in children, in order to better understand the underlying associations between these outcomes. Because this study found a significant relationship between electronic devices located specifically in the bedroom and estimated VO_2 Peak_ in a sample of children, and the presence of these electronic devices may influence markers of obesity, fitness, and sleep quality and quantity, sleep may play a mediating role in the relationship between the presence of electronic devices in children’s bedrooms and their CRF.

## Figures and Tables

**Table 1:** Descriptive statistics for the total sample and within sex groups (means and standard deviations).

	Total Sample (*N* = 106)	Girls (*n* = 56)	Boys (*n* = 50)
Age	9.7 (1.1)	9.7 (1.1)	9.8 (1.1)
BMI (kg/m^2^)	18.3 (2.9)	18.0 (3.0)	18.7 (2.8)
PE Composite	4.44 (2.9)	4.43 (3.0)	4.5 (2.9)
IB Composite	0.73 (1.0)	0.75 (1.1)	0.72 (0.83)
IH Composite	7.7 (3.7)	7.8 (4.1)	7.7 (3.2)
PACER Laps	31.6 (16.0)	29.6 (15.9)	34.0 (16.0)
Estimated VO_2 Peak_ (mL/kg/min)	45.9 (5.6)	45.2 (5.7)	46.7 (5.4)

BMI stands for Body Mass Index; PE Composite is the number of electronics personally owned; IB Composite is the number of personal electronics in the bedroom; IH Composite is the number of personal electronics in the home.

**Table 2:** Parameter estimates from hierarchical ridge regression models for the estimated maximal aerobic capacity (VO_2 Peak_) (mL/kg/min).

Outcome	Block	Predictor	b-Coefficient	95% C.I. ^1^	*p* Value
Estimated VO_2 Peak_	Block #1	PE Composite ^2^	−0.08	−0.45, 0.28	0.640
		IB Composite ^3^	**−1.29 ^†^**	−2.49, −0.10	0.034
		IH Composite ^4^	0.05	−0.27, 0.37	0.739
	Block #1 + Block #2	PE Composite	−0.01	−0.37, 0.36	0.964
		IB Composite	**−1.30 ^†^**	−2.46, −0.15	0.028
		IH Composite	0.03	−0.28, 0.34	0.847
		Boys ^6^	1.79	−0.31, 3.89	0.093
		Age	−0.07	−1.06, 0.93	0.893
		BMI ^5^	**−0.51 ^†^**	−0.89, −0.13	0.010
PACER Laps	Block #1	PE Composite	−0.19	−1.25, 0.86	0.713
		IB Composite	**−3.50 ^†^**	−6.91, −0.10	0.044
		IH Composite	0.20	−0.71, 1.11	0.661
	Block #1 + Block #2	PE Composite	−0.02	−1.11, 1.00	0.964
		IB Composite	**−3.70 ^†^**	−6.97, −0.41	0.028
		IH Composite	0.09	−0.79, 0.97	0.847
		Boys	**5.07**	−0.87, 11.01	0.093
		Age	−0.07	−1.06, 0.93	0.893
		BMI	**−1.43 ^†^**	−2.51, −0.36	0.010

^1^ 95% C.I. stands for 95% Confidence Interval; ^2^ PE Composite is the number of personal electronics personally owned; ^3^ IB Composite is the number of personal electronics in the bedroom; ^4^ IH Composite is the number of personal electronics in the home; ^5^ BMI stands for Body Mass Index; ^6^ The referent for sex is girls; bold and † denote statistical significance, *p* < 0.05.
